# Catechol-*O*-Methyltransferase Gene Polymorphism Modifies the Effect of Coffee Intake on Incidence of Acute Coronary Events

**DOI:** 10.1371/journal.pone.0000117

**Published:** 2006-12-27

**Authors:** Pertti Happonen, Sari Voutilainen, Tomi-Pekka Tuomainen, Jukka T. Salonen

**Affiliations:** 1 Department of Public Health, School of Public Health and Clinical Nutrition, University of Kuopio, Kuopio, Finland; 2 The Research Institute of Public Health, University of Kuopio, Kuopio, Finland; 3 Atherosclerosis Unit, Clinical Research Centre, University of Kuopio, Kuopio, Finland; 4 Oy Jurilab Ltd, Kuopio, Finland; Baylor College of Medicine, United States of America

## Abstract

**Background:**

The role of coffee intake as a risk factor for coronary heart disease (CHD) has been debated for decades. We examined whether the relationship between coffee intake and incidence of CHD events is dependent on the metabolism of circulating catecholamines, as determined by functional polymorphism of the catechol-*O*-methyltransferase (COMT) gene.

**Methodology/Principal Findings:**

In a cohort of 773 men who were 42 to 60 years old and free of symptomatic CHD at baseline in 1984–89, 78 participants experienced an acute coronary event during an average follow-up of 13 years. In logistic regression adjusting for age, smoking, family history of CHD, vitamin C deficiency, blood pressure, plasma cholesterol concentration, and diabetes, the odds ratio (90% confidence interval) comparing heavy coffee drinkers with the low activity COMT genotype with those with the high activity or heterozygotic genotypes was 3.2 (1.2–8.4). Urinary adrenaline excretion increased with increasing coffee intake, being over two-fold in heavy drinkers compared with nondrinkers (p = 0.008 for trend).

**Conclusions/Significance:**

Heavy coffee consumption increases the incidence of acute coronary events in men with low but not high COMT activity. Further studies are required to determine to which extent circulating catecholamines mediate the relationship between coffee intake and CHD.

## Introduction

Despite decades of research, controversy persists regarding the effects of coffee consumption on cardiovascular health [1], [2]. Coffee drinking is a prevalent habit worldwide and one constituent of coffee, caffeine, is probably the most frequently ingested pharmacologically active substance in the world [3]. Many of the known or suspected cardiovascular effects of coffee have been attributed to caffeine, but coffee is a mixture of hundreds of chemical substances, many of which have been shown to be pharmacologically active. For example, cafestol increases serum LDL cholesterol concentrations [4], caffeic acid and other polyphenols in coffee are potent antioxidants [5], and even decaffeinated coffee acutely increases blood pressure and muscle sympathetic nerve activity [6].

We have recently reported a U-shaped dose–response relationship between intake of caffeine-containing coffee and incidence of acute coronary events [7]; similar findings have been reported from at least two other studies [8], [9]. In order to gain further insight into the pathogenetic mechanisms, we hypothesized that the increased risk in heavy drinkers of caffeine-containing coffee is, at least partly, mediated by increased circulating catecholamine activity. Caffeine is a potent stimulator of plasma renin activity and adrenomedullary secretion; acute ingestion results in substantial increases in plasma concentrations of adrenaline and noradrenaline [10]. Tolerance to these acute humoral effects may develop in the course of one to four days of habitual consumption [11], but is not complete [12] and may be lost after abstinence for as little as 12 hours [13], [14].

To test this hypothesis, we examined whether the functional polymorphism of the catechol-*O*-methyltransferase (COMT) gene, resulting in several-fold differences in the metabolism of circulating catecholamines, modifies the effect of heavy consumption of caffeine-containing coffee on the risk of acute coronary events in a cohort of middle-aged eastern Finnish men initially free from symptomatic CHD.

## Methods

### Study design and population

The Kuopio Ischaemic Heart Disease Risk Factor Study is a population-based prospective follow-up study of 2682 men aged 42, 48, 54, or 60 years at the baseline examination carried out in 1984–89 [15]. The study was approved by the University of Kuopio Research Ethics Committee; all participants gave written informed consent. The study population was recruited in two cohorts. The present analysis is based on the latter cohort of 1516 men enrolled between 1986 and 1989. After exclusion of 353 men with prevalent CHD at baseline, as defined earlier [16], data on coffee consumption and smoking, as well as a deoxyribonucleic acid (DNA) sample, were available for 773 men.

### DNA Extraction and COMT Genotyping

COMT polymorphism is generated by the presence of guanine or adenine at nucleotide 475 encoding a valine or methionine at codon 158 and is recognized by the heat-shock protein 92 II restriction endonuclease. The polymorphism results in three different genotypes: homozygous for the low activity allele (methionine/methionine; *LL* genotype), homozygous for the high activity allele (valine/valine; *HH* genotype), and heterozygous (methionine/valine; *LH* genotype).

DNA was extracted from 10 mL of ethylenediaminetetraacetic-acid-anticoagulated venous blood using standard salting-out or phenol-chloroform assays. COMT genotypes were determined by restriction fragment length polymorphism analysis from the DNA by an investigator unaware of the phenotype. A 179 base-pair polymerase chain reaction product was generated using the forward 5′-CTG CTG GAG CTG GGG GCC GAC-3′ and reverse 5′-AGG TCT TCA GGA ATG C-3′ primers. The polymerase chain reaction product (5 µL) was treated with two units of heat-shock protein 92 II for 16 hours at 37°C and then analyzed by 2% agarose gel electrophoresis. The diagnostic bands were 179 (valine) and 139 (methionine).

### Assessment of Coffee Intake and Diet

Consumption of foods and beverages was assessed with an instructed and interview-checked 4-day food recording by household measures, including cups of coffee and tea [17]. The volume of the cup mainly used by each participant was estimated by showing photographs of four different cup sizes generally available. Dietary intake of foods and nutrients was calculated using NUTRICA software (National Public Health Institute, Turku, Finland).

### Measurement of Covariates and Catecholamine Excretion

The examination protocol and measurements have been described previously [15], [16]. Briefly, a participant was defined a current smoker, if he had ever smoked on a regular basis and had smoked within the past 30 days. The lifelong exposure to smoking was estimated as the product of years smoked and the number of cigarettes, cigars, and pipefuls smoked at the baseline examination. Diabetes was defined as self-reported diabetes mellitus or fasting blood glucose of 6.1 mM or more. Assessment of alcohol consumption, serum lipids, blood pressure, and body mass index [16], conditioning leisure-time physical activity [18], waist–hip ratio [19], and plasma vitamin C concentration [20] was carried out as described previously.

Urinary adrenaline and noradrenaline were purified with commercial phenylboronic acid columns and analyzed under isocratic conditions by reverse-phase liquid chromatography (for a detailed description, see [21]).

### Ascertainment of CHD Events

The collection of data on and classification of possible acute myocardial infarction and coronary death (here referred to as “acute coronary events”) until the end of 1992 is previously described [16], [22]. From 1993, data on acute coronary events were obtained by computer linkage to the national hospital discharge registry; diagnostic information was collected from the hospitals and classified using identical diagnostic criteria. There were no losses to follow-up. In case of multiple events in the same participant, the first event was considered the endpoint.

### Statistical Analysis

All analyses were performed with SPSS version 11.5 (SPSS Inc., Chicago, IL). Average daily coffee intake was divided into four categories: 0 (non-drinkers), 1 to 375 mL (light drinkers), 376 to 813 mL (moderate drinkers), and 814 mL and over (heavy drinkers). (Since the coffee content in the most common cup size in this population is 125 mL, these coffee intake categories approximately correspond to less than 3 cups, 3 to 6.5 cups, and more than 6.5 cups per day, respectively.) To evaluate biologic interaction (departures from additivity), a single composite variable with six joint-exposure categories was created; the COMT *LH* and *LL* genotypes were combined into a single category and coffee non-drinkers were combined with light drinkers. Logistic regression was used to assess the association between the composite coffee intake–COMT variable and acute coronary events; heavy coffee drinkers with either the *HH* or *LH* genotype were used as the reference category. Continuous covariates without a meaningful zero value were centered around their respective means. Statistical inference was based on 90% Wald confidence intervals or two-sided p-values.

In a subgroup of 258 participants with urinary catecholamine measurements, we performed a supplemental linear regression analysis of urinary adrenaline and noradrenaline excretion in categories of coffee intake. Data on catecholamine excretion according to COMT genotype were not available.

## Results

Baseline characteristics according to coffee intake are shown in Table 1 and according to COMT genotype in Table 2. In comparison with light to moderate drinkers, heavy coffee drinkers were more likely to be smokers, had a higher energy and saturated fat intake but lower leisure-time physical activity level, and their LDL cholesterol concentrations were higher. Compared with nondrinkers, coffee drinkers were more likely to be smokers and to have a family history of CHD; had lower leisure-time physical activity levels, a higher body mass index and waist–hip ratio, higher systolic and diastolic blood pressure; and higher LDL cholesterol concentration. Participants with the COMT *HH* genotype were less likely to be diabetic and had lower levels of leisure-time physical activity than those with the other two genotypes; systolic blood pressure was marginally higher in the *LL* genotype.

**Table 1:**
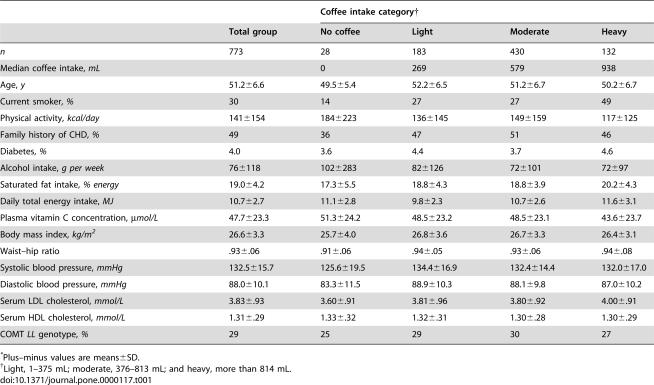
Characteristics of the study participants, according to their average daily consumption of coffee.*

		Coffee intake category†			
	Total group	No coffee	Light	Moderate	Heavy
*n*	773	28	183	430	132
Median coffee intake, *mL*		0	269	579	938
Age, *y*	51.2±6.6	49.5±5.4	52.2±6.5	51.2±6.7	50.2±6.7
Current smoker, *%*	30	14	27	27	49
Physical activity, *kcal/day*	141±154	184±223	136±145	149±159	117±125
Family history of CHD, *%*	49	36	47	51	46
Diabetes, *%*	4.0	3.6	4.4	3.7	4.6
Alcohol intake, *g per week*	76±118	102±283	82±126	72±101	72±97
Saturated fat intake, *% energy*	19.0±4.2	17.3±5.5	18.8±4.3	18.8±3.9	20.2±4.3
Daily total energy intake, *MJ*	10.7±2.7	11.1±2.8	9.8±2.3	10.7±2.6	11.6±3.1
Plasma vitamin C concentration, *µmol/L*	47.7±23.3	51.3±24.2	48.5±23.2	48.5±23.1	43.6±23.7
Body mass index, *kg/m^2^*	26.6±3.3	25.7±4.0	26.8±3.6	26.7±3.3	26.4±3.1
Waist–hip ratio	.93±.06	.91±.06	.94±.05	.93±.06	.94±.08
Systolic blood pressure, *mmHg*	132.5±15.7	125.6±19.5	134.4±16.9	132.4±14.4	132.0±17.0
Diastolic blood pressure, *mmHg*	88.0±10.1	83.3±11.5	88.9±10.3	88.1±9.8	87.0±10.2
Serum LDL cholesterol, *mmol/L*	3.83±.93	3.60±.91	3.81±.96	3.80±.92	4.00±.91
Serum HDL cholesterol, *mmol/L*	1.31±.29	1.33±.32	1.32±.31	1.30±.28	1.30±.29
COMT *LL* genotype, *%*	29	25	29	30	27

* Plus–minus values are means±SD.

† Light, 1–375 mL; moderate, 376–813 mL; and heavy, more than 814 mL.

**Table 2:**
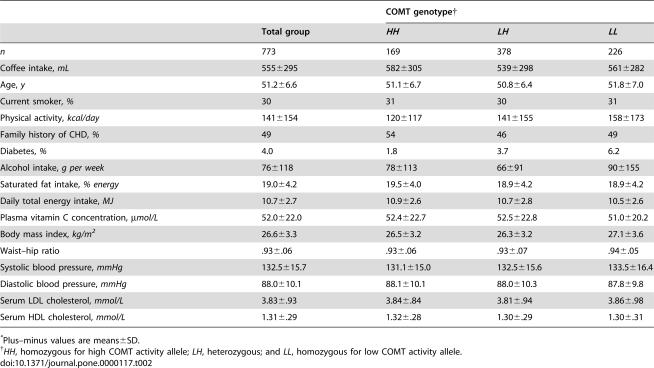
Characteristics of the study participants, according to their COMT genotype.*

		COMT genotype†		
	Total group	*HH*	*LH*	*LL*
*n*	773	169	378	226
Coffee intake, *mL*	555±295	582±305	539±298	561±282
Age, *y*	51.2±6.6	51.1±6.7	50.8±6.4	51.8±7.0
Current smoker, *%*	30	31	30	31
Physical activity, *kcal/day*	141±154	120±117	141±155	158±173
Family history of CHD, *%*	49	54	46	49
Diabetes, *%*	4.0	1.8	3.7	6.2
Alcohol intake, *g per week*	76±118	78±113	66±91	90±155
Saturated fat intake, *% energy*	19.0±4.2	19.5±4.0	18.9±4.2	18.9±4.2
Daily total energy intake, *MJ*	10.7±2.7	10.9±2.6	10.7±2.8	10.5±2.6
Plasma vitamin C concentration, *µmol/L*	52.0±22.0	52.4±22.7	52.5±22.8	51.0±20.2
Body mass index, *kg/m^2^*	26.6±3.3	26.5±3.2	26.3±3.2	27.1±3.6
Waist–hip ratio	.93±.06	.93±.06	.93±.07	.94±.05
Systolic blood pressure, *mmHg*	132.5±15.7	131.1±15.0	132.5±15.6	133.5±16.4
Diastolic blood pressure, *mmHg*	88.0±10.1	88.1±10.1	88.0±10.3	87.8±9.8
Serum LDL cholesterol, *mmol/L*	3.83±.93	3.84±.84	3.81±.94	3.86±.98
Serum HDL cholesterol, *mmol/L*	1.31±.29	1.32±.28	1.30±.29	1.30±.31

* Plus–minus values are means±SD.

† *HH*, homozygous for high COMT activity allele; *LH*, heterozygous; and *LL*, homozygous for low COMT activity allele.

In the supplemental analysis of urinary catecholamine excretion among 258 participants, the amount of adrenaline excreted was over two-fold in the heavy coffee intake category compared with non-drinkers (p = 0.008 for trend); for noradrenaline, the respective difference was 30% (p = 0.039 for trend).

**Figure 1:**
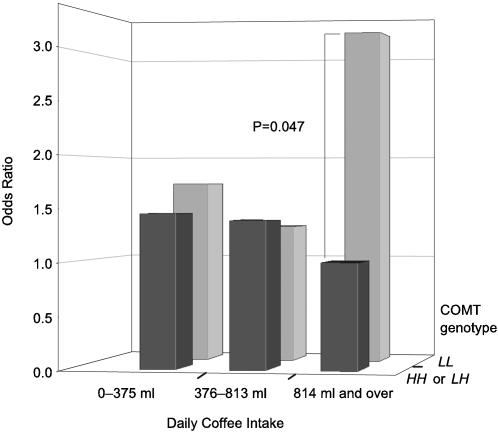
Odds ratios of acute coronary events in 773 men initially free from CHD by joint categories of coffee intake and COMT genotype; predicted from a logistic model with age, smoking, family history of CHD, plasma vitamin C concentration, systolic blood pressure, serum HDL and LDL cholesterol concentration, and diabetes as covariates.

A total of 78 acute coronary events were identified during an average follow-up of 13 years. The cumulative incidence rate in other categories of coffee intake was relatively stable (around 10%) but clearly increased in heavy drinkers with the COMT *LL* genotype (Table 3). After adjustment for age, packyears of smoking, family history of CHD, and vitamin C deficiency, the odds ratio (90% confidence interval) comparing heavy drinkers in the *LL* genotype with heavy drinkers in the other two genotypes was 3.25 (1.29–8.16). Further adjustment for systolic blood pressure, serum LDL and HDL cholesterol concentration, and diabetes minimally decreased the odds ratio estimate to 3.21 (1.22–8.42). In the combined *HH* or *LH* category, the adjusted odds ratios for light and moderate coffee drinkers were 1.45 (0.64–3.27) and 1.39 (0.67–2.88), respectively; the corresponding odds ratios in the *LL* genotype were 1.73 (0.68–4.42) and 1.31 (0.58–2.99), using the heavy drinkers with either the COMT *HH* or *LH* genotype as the reference category (Figure 1). Adjustment for physical activity; intake of alcohol, saturated fat, or total energy; or body mass index and waist–hip ratio did not change the odds ratio estimates appreciably or contribute to model fit.

**Table 3:**
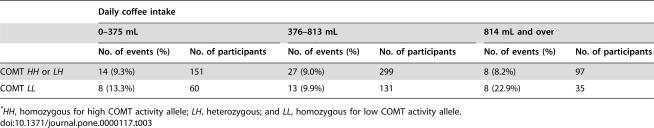
Distribution of acute coronary events in the study population during an average follow-up of 13 years, according to the joint coffee intake–COMT genotype categories.*

	Daily coffee intake					
	0–375 mL		376–813 mL		814 mL and over	
	No. of events (%)	No. of participants	No. of events (%)	No. of participants	No. of events (%)	No. of participants
COMT *HH* or *LH*	14 (9.3%)	151	27 (9.0%)	299	8 (8.2%)	97
COMT *LL*	8 (13.3%)	60	13 (9.9%)	131	8 (22.9%)	35

* *HH*, homozygous for high COMT activity allele; *LH*, heterozygous; and *LL*, homozygous for low COMT activity allele.

## Discussion

In this population-based cohort of middle-aged men initially free from symptomatic CHD followed-up for an average of 13 years, we found a relationship between consumption of caffeine-containing coffee and the incidence of fatal or nonfatal CHD events that is highly dependent on COMT genotype. In men who were either homozygous for the high COMT activity allele or heterozygotes, heavy coffee intake did not increase the incidence of acute coronary events. In men who were homozygous for the low-activity COMT allele, however, heavy coffee intake was associated with a considerably higher incidence of acute coronary events. The relative excess in CHD incidence in the low-activity genotype was over two-fold among drinkers of more than 6.5 cups of coffee per day, after adjustment for age, smoking, family history of CHD, plasma vitamin C concentration, systolic blood pressure, serum LDL and HDL cholesterol concentration, and diabetes.

We are not aware of any previous studies that have addressed the modification of the coffee–CHD relationship by COMT activity. Tens of studies investigating the relationship between coffee consumption and the incidence of CHD have been carried out, but the results remain inconsistent. Several large studies carried out in the United States have found no appreciable increase in CHD risk with increasing coffee consumption [23]–[25]. Two earlier U.S. studies, however, reported a two-fold risk of myocardial infarction [26] or CHD [27] in men drinking 5 cups of coffee or more; the risk was three-fold among those drinking 10 cups or more [26], compared with non-drinkers.

The findings have been in part dependent on the type of study: case–control studies have generally reported higher effect estimates than cohort studies [1]. In studies published more recently, the discrepancy between cohort and case–control studies persists [28]–[30]. As an explanation for the discrepancy between study types it has been suggested that coffee drinking has mainly acute or short-term effects, which cohort studies with extended follow-up periods would be likely to miss [27]. In general, the inconsistency between studies has been attributed to differences in coffee brews, study populations, range of coffee intake, confounding by smoking or other lifestyle factors, and measurement inaccuracy.

Two recent studies have observed a U-shaped pattern between coffee consumption and CHD incidence [7], [9], suggesting that the relationship between coffee intake and CHD is more complex than previously recognized and offering yet another potential explanation for the contradictory findings in the literature. In both studies, the J- or U-shaped association persisted after adjustment for known risk factors for CHD, such as hypertension, high LDL cholesterol concentration, and diabetes, which could partially mediate the effects of coffee intake. In our previous study [7], the association was also independent of the brewing method of coffee, which has long been offered as a likely explanation for increased risk among heavy drinkers of non-filtered coffee [31]. In addition, the association was stronger during the first years of follow-up, which indicates that coffee intake is more relevant with respect to the acute rather than chronic processes related to coronary disease.

Caffeine is metabolized by the polymorphic cytochrome P450 1A2 (CYP1A2) enzyme. Recently Cornelis and co-workers determined whether CYP1A2 genotype modifies the association between intake of caffeinated coffee and the risk of nonfatal myocardial infarction [32]. They found that intake of coffee was associated with an increased risk of myocardial infarction only among individuals with slow caffeine metabolism and suggested that caffeine plays a role in this association.

These findings led us to consider novel explanations behind the dose–response relationship between coffee consumption and CHD incidence. Coffee and caffeine have been shown to stimulate adrenomedullary secretion, resulting in raised levels of circulating catecholamines, adrenaline in particular [10], [33]. In a subgroup of the present study population with urinary catecholamine excretion measured at baseline, we also observed a marked increase in adrenaline excretion and some increase in the excretion of noradrenaline with increasing coffee intake. Increases in circulating catecholamine levels and cortisol are characteristic of the response to stress, both psychological and physiological, and caffeine has been shown to potentiate stress-related increases in plasma adrenaline and cortisol concentrations, both in habitual and light consumers [12]. Stress in turn is a recognized risk factor for CHD. Furthermore, stress appears to be more strongly related to the rapid and more transient processes (such as rupture of atherosclerotic plaques with subsequent platelet activation, thrombus formation, and vasospasm) that lead to acute coronary syndromes rather than to the long-term atherosclerotic process [34]. On the other hand, treatment with beta-adrenoceptor blocking agents has been shown to effectively reduce the risk of acute coronary syndromes during acute periods of stress, such as surgical stress [35], which is characterized by elevated adrenaline and cAMP levels [36].

Hence, we hypothesized that heavy intake of caffeine-containing coffee induces a “chemical stress” and that the effect of this stress response on CHD incidence is more evident in those whose catecholamine metabolism is slower than usual and the mechanisms of tolerance may be overwhelmed. The main enzyme responsible for the metabolism of circulating catecholamines is COMT [37]. COMT activity shows functional polymorphism determined by the COMT gene: those who are homozygous for the low activity allele (about 25% of a Caucasian population) have only one-half to one-fourth of the enzyme activity of the other genetic variants [38]. These differences in COMT activity by genotype are likely to be more pronounced in heavy consumers of coffee, because caffeic acid directly inhibits COMT activity [39] and caffeine inhibits adenosine deaminase [40], resulting in a shift in homocysteine metabolism to the direction of *S*-adenosyl-homocysteine and consequent further inhibition of COMT activity [41]. Our finding of a 2.2 fold increase in the incidence of acute coronary events among heavy coffee drinkers in the low-activity COMT category, compared with the other two COMT categories, is highly compatible with this hypothesis.

Our study is limited by the small study cohort and the fact that only 78 CHD events were observed during an average follow-up of 13 years, resulting in imprecise effect estimates reflected as wide confidence intervals. Nevertheless, we were able to control for the most important potential confounding factors and still the difference in CHD incidence between the COMT activity categories among heavy coffee drinkers persisted. Residual confounding alone is unlikely to explain the remaining excess incidence. Another major limitation of our study is restriction of the study population to men only. Female hormones may influence catecholamine pharmacodynamics [42] and thus our findings are not necessarily applicable to women.

In conclusion, we suggest that circulating catecholamines play a role as mediators of the effect of heavy coffee intake on the risk of CHD events. The elevated risk may be more pronounced in or even restricted to those whose catecholamine metabolism is slower than usual. Further research is required to confirm or refute our hypothesis.

## References

[1] Greenland S (1993). A meta-analysis of coffee, myocardial infarction, and coronary death.. Epidemiology.

[2] Gyntelberg F, Hein HO, Suadicani P, Sørensen H (1995). Coffee consumption and risk of ischaemic heart disease–a settled issue?. J Intern Med.

[3] Nawrot P, Jordan S, Eastwood J, Rotstein J, Hugenholtz A (2003). Effects of caffeine on human health.. Food Addit Contam.

[4] Boekschoten MV, Engberink MF, Katan MB, Schouten EG (2003). Reproducibility of the serum lipid response to coffee oil in healthy volunteers.. Nutr J.

[5] Svilaas A, Sakhi AK, Andersen LF, Svilaas T, Ström EC (2004). Intakes of antioxidants in coffee, wine, and vegetables are correlated with plasma carotenoids in humans.. J Nutr.

[6] Corti R, Binggeli C, Sudano I, Spieker L, Hänseler E (2002). Coffee acutely increases sympathetic nerve activity and blood pressure independently of caffeine content: role of habitual versus nonhabitual drinking.. Circulation.

[7] Happonen P, Voutilainen S, Salonen JT (2004). Coffee drinking is dose-dependently related to the risk of acute coronary events in middle-aged men.. J Nutr.

[8] Woodward M, Tunstall-Pedoe H (1999). Coffee and tea consumption in the Scottish Heart Health Study follow up: conflicting relations with coronary risk factors, coronary disease, and all cause mortality.. J Epidemiol Community Health.

[9] Panagiotakos DB, Pitsavos C, Chrysohoou C, Kokkinos P, Toutouzas P (2003). The J-shaped effect of coffee consumption on the risk of developing acute coronary syndromes: the CARDIO2000 case-control study.. J Nutr.

[10] Robertson D, Frölich JC, Carr RK, Watson JT, Hollifield JW (1978). Effects of caffeine on plasma renin activity, catecholamines and blood pressure.. N Engl J Med.

[11] Robertson D, Wade D, Workman R, Woosley RL, Oates JA (1981). Tolerance to the humoral and hemodynamic effects of caffeine in man.. J Clin Invest.

[12] Lane JD, Adcock RA, Williams RB, Kuhn CM (1990). Caffeine effects on cardiovascular and neuroendocrine responses to acute psychosocial stress and their relationship to level of habitual caffeine consumption.. Psychosom Med.

[13] Whitsett TL, Manion CV, Christensen HD (1984). Cardiovascular effects of coffee and caffeine.. Am J Cardiol.

[14] Pincomb GA, Lovallo WR, Passey RB, Wilson MF (1988). Effect of behavior state on caffeine's ability to alter blood pressure.. Am J Cardiol.

[15] Salonen JT (1988). Is there a continuing need for longitudinal epidemiologic research? The Kuopio Ischaemic Heart Disease Risk Factor Study.. Ann Clin Res.

[16] Salonen JT, Nyyssönen K, Korpela H, Tuomilehto J, Seppänen R (1992). High stored iron levels are associated with excess risk of myocardial infarction in Eastern Finnish men.. Circulation..

[17] Ihanainen M, Salonen R, Seppänen R, Salonen JT (1989). Nutrition data collection in the Kuopio Ischaemic Heart Disease Risk Factor Study: Nutrient intake of middle-aged Eastern Finnish men.. Nutr Res.

[18] Lakka TA, Venäläinen JM, Rauramaa R, Salonen R, Tuomilehto J (1994). Relation of leisure-time physical activity and cardiorespiratory fitness to the risk of acute myocardial infarction.. N Engl J Med.

[19] Lakka HM, Laaksonen DE, Lakka TA, Niskanen LK, Kumpusalo E (2002). The metabolic syndrome and total and cardiovascular disease mortality in middle-aged men.. JAMA.

[20] Nyyssönen K, Parviainen MT, Salonen R, Tuomilehto J, Salonen JT (1997). Vitamin C deficiency and risk of myocardial infarction: prospective population study of men from eastern Finland.. BMJ.

[21] Salonen JT, Salonen R, Ihanainen M, Parviainen M, Seppanen R (1988). Blood pressure, dietary fats, and antioxidants.. Am J Clin Nutr.

[22] Tuomilehto J, Arstila M, Kaarsalo E, Kankaanpää J, Ketonen M (1992). Acute myocardial infarction (AMI) in Finland—baseline data from the FINMONICA AMI register in 1983–1985.. Eur Heart J.

[23] Wilson PWF, Garrison RJ, Kannel WB, McGee DL, Castelli WP (1989). Is coffee consumption a contributor to cardiovascular disease? Insights from the Framingham study.. Arch Intern Med.

[24] Grobbee DE, Rimm EB, Giovannucci E, Colditz G, Stampfer M (1990). Coffee, caffeine, and cardiovascular disease in men.. N Engl J Med.

[25] Willett WC, Stampfer MJ, Manson JE, Colditz GA, Rosner BA (1996). Coffee consumption and coronary heart disease in women. A ten-year follow-up.. JAMA.

[26] Rosenberg L, Palmer JR, Kelly JP, Kaufman DW, Shapiro S (1988). Coffee drinking and nonfatal myocardial infarction in men under 55 years of age.. Am J Epidemiol.

[27] LaCroix AZ, Mead LA, Liang KY, Thomas CB, Pearson TA (1986). Coffee consumption and the incidence of coronary heart disease.. N Engl J Med.

[28] Kleemola P, Jousilahti P, Pietinen P, Vartiainen E, Tuomilehto J (2000). Coffee consumption and the risk of coronary heart disease and death.. Arch Intern Med.

[29] Tavani A, Bertuzzi M, Negri E, Sorbara L, La Vecchia C (2001). Alcohol, smoking, coffee and risk of non-fatal acute myocardial infarction in Italy.. Eur J Epidemiol.

[30] Hammar N, Andersson T, Alfredsson L, Reuterwall C, Nilsson T (2003). Association of boiled and filtered coffee with incidence of first nonfatal myocardial infarction: the SHEEP and the VHEEP study.. J Intern Med.

[31] Thelle DS (1995). Coffee, tea and coronary heart disease.. Curr Opin Lipidol.

[32] Cornelis MC, El-Sohemy A, Kabagambe EK, Campos H (2006). Coffee, CYP1A2 genotype, and risk of myocardial infarction.. JAMA.

[33] Smits P, Pieters G, Thien T (1986). The role of epinephrine in the circulatory effects of coffee.. Clin Pharmacol Ther.

[34] Gidron Y, Gilutz H, Berger R, Huleihel M (2002). Molecular and cellular interface between behavior and acute coronary syndromes.. Cardiovasc Res.

[35] Madsen SN, Fog-Moller F, Christiansen C, Vester-Andersen T, Engquist A (1978). Cyclic AMP, adrenaline and noradrenaline in plasma during surgery.. Br J Surg.

[36] Stevens RD, Burri H, Tramer MR (2003). Pharmacologic myocardial protection in patients undergoing noncardiac surgery: a quantitative systematic review.. Anesth Analg.

[37] Eisenhofer G, Huynh TT, Hiroi M, Pacak K (2001). Understanding catecholamine metabolism as a guide to the biochemical diagnosis of pheochromocytoma.. Rev Endocr Metab Disord.

[38] Syvänen AC, Tilgmann C, Rinne J, Ulmanen I (1997). Genetic polymorphism of catechol-O-methyltransferase (COMT): correlation of genotype with individual variation of S-COMT activity and comparison of the allele frequencies in the normal population and parkinsonian patients in Finland.. Pharmacogenetics.

[39] Kumada Y, Naganawa H, Iinuma H, Matsuzaki M, Takeuchi T (1976). Dehydrodicaffeic acid dilactone, an inhibitor of catechol-O-methyl transferase.. J Antibiot (Tokyo).

[40] Saboury AA, Divsalar A, Ataie G, Amanlou M, Moosavi-Movahedi AA (2003). Inhibition study of adenosine deaminase by caffeine using spectroscopy and isothermal titration calorimetry.. Acta Biochim Pol.

[41] Zhu BT (2002). On the mechanism of homocysteine pathophysiology and pathogenesis: a unifying hypothesis.. Histol Histopathol.

[42] Brandin L, Bergström G, Manhem K, Gustafsson H (2003). Oestrogen modulates vascular adrenergic reactivity of the spontaneously hypertensive rat.. J Hypertens.

